# Supplementary material for: The critical role of infection prevention overlooked in Ethiopia, only one-half of health-care workers had safe practice: A systematic review and meta-analysis
Source: PLoS One. 2021 Jan 14;16(1):e0245469. doi: 10.1371/journal.pone.0245469 (PMC7808611; doi:10.1371/journal.pone.0245469)
Supplement: Attachment — Submitted filename: My comments.docx [file pone.0245469.s005.docx]

Ref: PONE-D-20-11122

**The critical role of infection prevention overlooked in Ethiopia, only one-half of health-care workers had safe practice: A Systematic Review and Meta-Analysis**

**Dear Editor**

Thank for you for the opportunity to review this manuscript. The study reviews the prevalence of safe prevention practices and the associated factors among healthcare workers in Ethiopia.

**General comments:**

This study is of interest to the readers, particularly those seeking to understand the situation of safe infection prevention in Ethiopia and its drivers.

The authors calculated the pooled prevalence from the prevalence of safe infection prevention practices reported by healthcare workers. In my opinion, the method would more perfect in estimating the awareness/knowledge of health care workers rather their practices.

What is the difference between “safe infection prevention” and “infection prevention” throughout the document? In some context, the words bring confusion than clarity.

Some repetitive phrases, grammatical error and typos to correct throughout the document need to be addressed eg page 8Ln12, pg10Ln51, pg10Ln66, 75, 79, 232

**Specific areas:**

**Abstract**

In which direction these factors were associated with safe prevention practices? Were they risk or protective factors?

**Background**

The authors highlighted the release of the second edition of National infection prevention publication in 2012. What are the proposed components of infection prevention practices as per this document in the country and how do they relate to the components assessed by the authors?

**Methodology**

The authors reported 10 studies qualified for systematic review and meta-analysis based on inclusion and exclusion criteria. Since being both a quantitative and qualitative was not an inclusion criteria; I failed to figure out the number of studies reviewed per study design: quantitative and qualitative. Were all qualified studies both quantitative and qualitative?

**Outcome of the study**

The definition of the primary outcome and how it was computed don't match, the computation gives an impression of the awareness/knowledge of the practices and not how they practice.

**Results**

Table 1: what were the components assessed by for the Bekele I et al, Hussen SH., et al studies?

Table 3: What is the reasoning behind the cut-off points in sample size and publication year variables? (Was there a new practise introduced before or after 2015?)

**Discussion**

How does this pooled prevalence compare to other findings elsewhere in Africa, for example.

**Limitation**

Most of the studies reviewed by the authors did not cover a good range of components of infection prevention practices. Could this be a limitation to keep in mind when presenting the obtained pooled prevalence?

The authors did not mitigate any of the limitations of the study

**References**

Include access date for web-based references.e.g. page32Ln391
